# Supplementary material for: Association between oral microbiome and seven types of cancers in East Asian population: a two-sample Mendelian randomization analysis
Source: Front Mol Biosci. 2023 Nov 21;10:1327893. doi: 10.3389/fmolb.2023.1327893 (PMC10702768; doi:10.3389/fmolb.2023.1327893)
Supplement: Supplementary file 1 [file DataSheet1.ZIP › Supplementary Materials/MR plots of saliva/saliva═╝/colorectal cancer/pheno.673_to_colorectal cancer_forest.pdf]

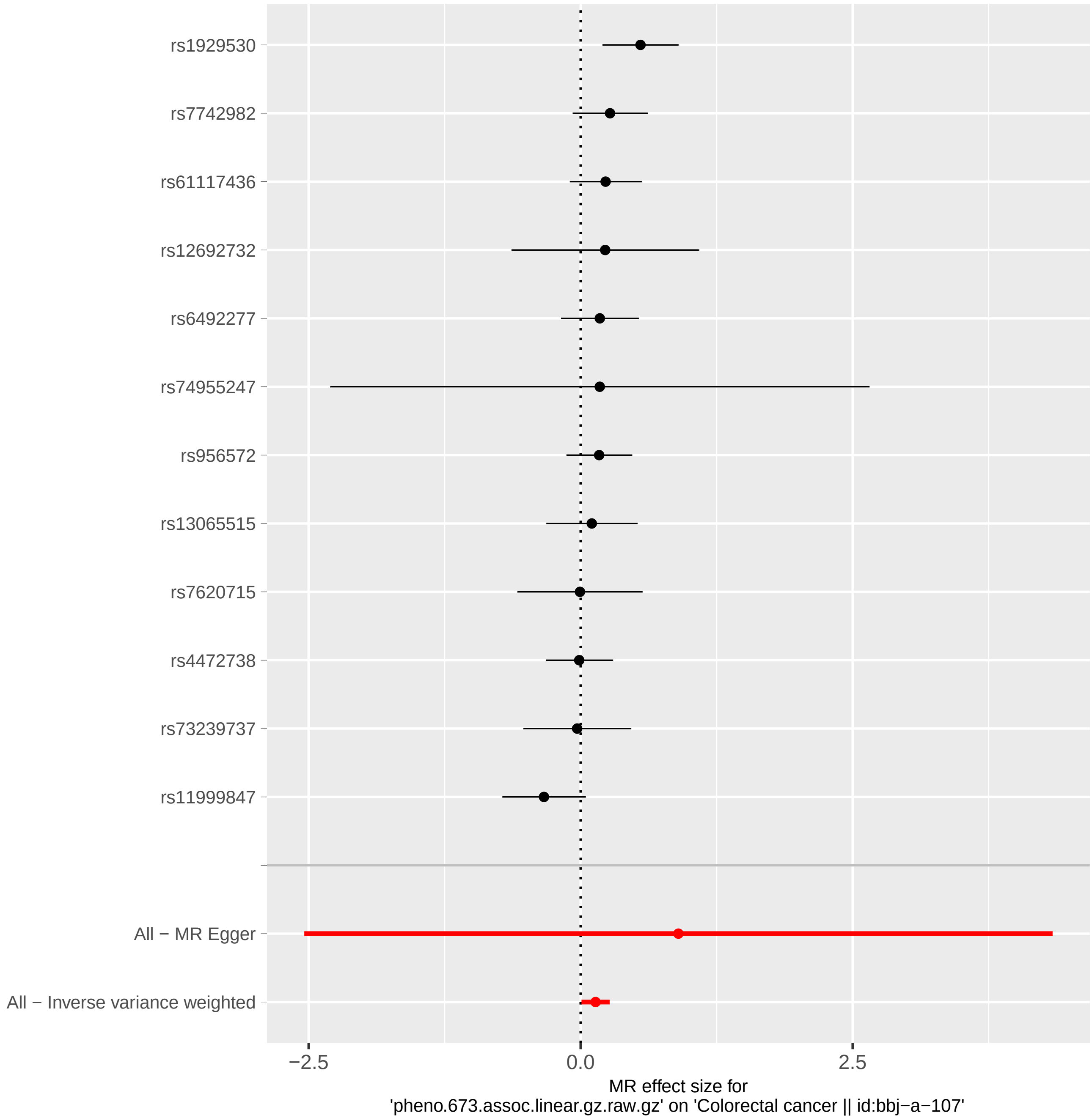
rs1929530
rs7742982
rs61117436
rs12692732
rs6492277
rs74955247
rs956572
rs13065515
rs7620715
rs4472738
rs73239737
rs11999847
All − MR Egger
All − Inverse variance weighted
−2.5
0.0
2.5
MR effect size for
'pheno.673.assoc.linear.gz.raw.gz' on 'Colorectal cancer || id:bbj−a−107'
